# Effects of nitric oxide on the GABA, polyamines, and proline in tea (*Camellia sinensis*) roots under cold stress

**DOI:** 10.1038/s41598-020-69253-y

**Published:** 2020-07-22

**Authors:** Yuhua Wang, Fei Xiong, Shouhua Nong, Jieren Liao, Anqi Xing, Qiang Shen, Yuanchun Ma, Wanping Fang, Xujun Zhu

**Affiliations:** 1 College of Horticulture, Nanjing Agricultural University, No.1 Weigang, Nanjing, 210095 Jiangsu China; 2 Institute of Tea Sciences, Guizhou Academy of Agricultural Sciences, Guiyang, 417100 China

**Keywords:** Plant molecular biology, Plant physiology, Plant stress responses

## Abstract

Tea plant often suffers from low temperature induced damage during its growth. How to improve the cold resistance of tea plant is an urgent problem to be solved. Nitric oxide (NO), γ-aminobutyric acid (GABA) and proline have been proved that can improve the cold resistance of tea plants, and signal transfer and biosynthesis link between them may enhance their function. NO is an important gas signal material in plant growth, but our understanding of the effects of NO on the GABA shunt, proline and NO biosynthesis are limited. In this study, the tea roots were treated with a NO donor (SNAP), NO scavenger (PTIO), and NO synthase inhibitor (L-NNA). SNAP could improve activities of arginine decarboxylase, ornithine decarboxylase, glutamate decarboxylase, GABA transaminase and Δ1-pyrroline-5-carboxylate synthetase and the expression level of related genes during the treatments. The contents of putrescine and spermidine under SNAP treatment were 45.3% and 37.3% higher compared to control at 24 h, and the spermine content under PTIO treatment were 57.6% lower compare to control at 12 h. Accumulation of proline of SNAP and L-NNA treatments was 52.2% and 43.2% higher than control at 48 h, indicating other pathway of NO biosynthesis in tea roots. In addition, the NO accelerated the consumption of GABA during cold storage. These facts indicate that NO enhanced the cold tolerance of tea, which might regulate the metabolism of the GABA shunt and of proline, associated with NO biosynthesis.

## Introduction

Tea plant is an important economic crop which is widely planted in many regions of China. Adverse environmental conditions, mainly cold stress, impose major limitations on the suitable geographical locations for tea growth and impact both in tea production and quality^1^.

The GABA is a non-protein amino acid, C_4_H_9_NO_2_^3^, and is widely distributed in nature among prokaryotes and eukaryotes as an important free amino acid. Some special treatments of fresh tea leaves, such as charging the nitrogen and removing oxygen can result in accumulation of GABA^2^. It is widely known as a neurotransmitter in the sympathetic nervous system^4,5^. Moreover, previous study also revealed its function on improving cold tolerance of tea plant^6^. There are several GABA metabolism pathways, including glutamate catalyzed by glutamate decarboxylase (GAD) and the reversible conversion of GABA to succinic semialdehyde by GABA transaminase (GABA-T) followed by irreversible oxidization of succinic semialdehyde. Polyamines (PAs) include spermidine (Spd), spermine (Spm) and their diamine obligate precursor putrescine (Put)^3,7^, PAs catabolism can provide raw materials for GABA synthesis which means PAs degradation pathway is an important component of the GABA biosynthesis pathway^8^. The Put and Spd are catalyzed by diamine oxidase and polyamine oxidase, respectively^3^. PAs are widely present in plants, and the enzymes related to their biosynthesis have strong connections with environmental stresses such as cold, heat, salt and drought stress^9,10^. The expression and activity of PAs biosynthetic enzymes play an important role in the accumulation of PAs^11^. According to Liao et al.^3^, ornithine decarboxylase (ODC) and arginine decarboxylase (ADC) convert ornithine and arginine to produce Put. The spermidine synthase (SPDS) catalyzes production of Spd from Put, and spermine synthase (SPMS) catalyzes Spd to Spm. In addition, PAs are related to NO biosynthesis to some extent in specific tissues in *Arabidopsis* seedlings^12^.

NO is widely distributed in plants as a gas signal material, which can induce numerous processes, including expression of defense genes, programmed cell death, stomatal closure and root development^13,14^. It is reported that when plants suffer from cold or chilling stress, the endogenous NO levels rise, and plants which are more resistant to cold stress can accumulate more NO^15,16^. Application of exogenous NO has been reported to help retard cold injury in maize, wheat and tomato^17–20^. There are many sources of NO in plants, mainly mediated by nitric oxide synthase (NOS) and nitrate reductase (NR). NO was produced by using L-arginine as substrate and NADPH as electron donor^21^. Sun et al.^22^ found that lipopolysaccharide (LPS) mainly used arginine to mediate NO production to inducing plant resistance. Arginine or its derivatives are potential sources of NO in *Arabidopsis* and reducing arginase activity can resulted in a large amount of arginine conversion to NO^23^. These studies show that the NOS may be essential for plants.

Accumulation of proline is believed to be closely related to environmental stress^24^. When plants face various abiotic stresses such as cold, drought and salinity, proline can accumulate rapidly^25,26^. In addition, the NO content is closely associated with the proline content^27,28^. The biosynthesis of proline has two components: glutamate and ornithine pathways. Delauney et al.^29^ found that the glutamate pathway dominated proline synthesis under osmotic stress or low NO conditions. The glutamate pathway suggests that glutamate can be continuously reduced in two steps to synthesize proline, with P5C the most important intermediate product. The key enzyme in this reaction is Δ1-pyrroline-5-carboxylate synthetase (P5CS) ^30^. In the ornithine pathway, ornithine is transaminated to GSA or α-keto-δ-aminovalerate by δ-ornithine-amino-transferase (δ-OAT), then the GSA spontaneously cyclizes to P5C, which is converted to proline by P5CR. The α-keto-δ-aminovalerate spontaneously cyclizes to pyrroline 2-carboxylate, which is converted to proline by pyrroline 2-carboxylate reductase^31^.

Glutamate is one of the precursors of GABA and proline^32^, and exhibits a strong connection with ornithine and arginine, which are precursors of proline and NO, respectively. We expected to clarify the role of NO, GABA and proline in the cold resistance of tea plants and find their internal connection. However, there are no relevant studies in this field of tea plants. In this study, we explored the nitric oxide (NO) affected the γ-aminobutyric acid (GABA) shunt and accumulation of proline to improve the cold resistance of tea plants. Our research aims to preliminary explore their relationship under low temperature conditions and their contribution to improving the cold resistance of tea plants.

## Results

### GABA metabolism

**Figure 1:**
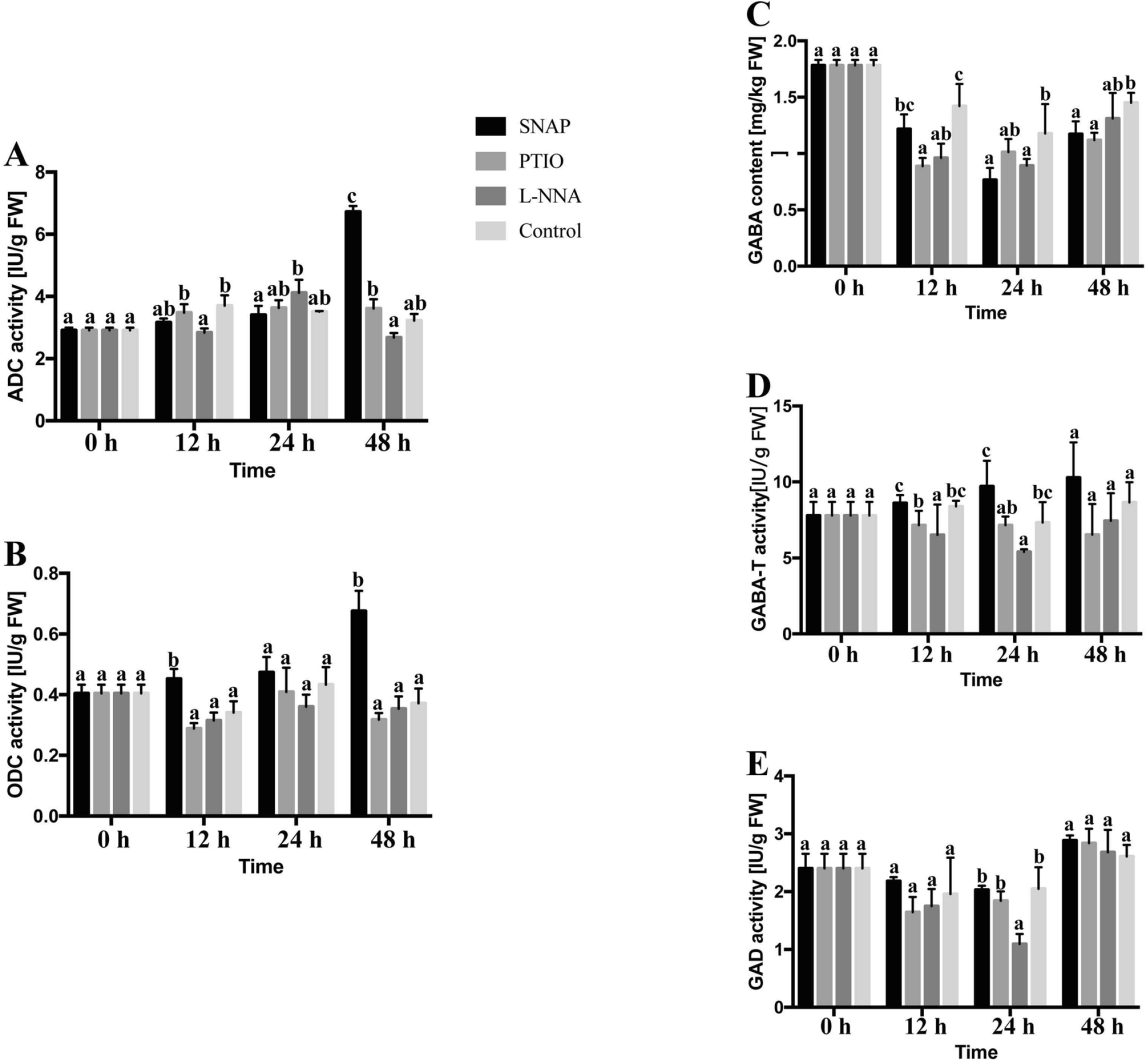
Changes of GABA metabolism related enzyme activities (ADC, ODC, GABA-T and GAD) and GABA content during the different treatments. Values are means ± SE (n = 3), p ≤ 0.05.

The treatments with SNAP, PTIO and L-NNA were accompanied by cold stress. The ADC activity (Fig. 1A) of PTIO treatment and the control showed slight increases within 24 h. In addition, the ADC activity of SNAP treatment was 108%, 150% and 86% higher than control, L-NNA and PTIO treatments at 48 h, respectively.

The ODC activity (Fig. 1B) with SNAP treatment was higher than control at three time points: 32.9%, 9.5% and 81.7% higher at 12 h, 24 h and 48 h, respectively. The PTIO treatment showed the same trends as the control, but with slight lower levels.

The GAD activity (Fig. 1E) showed a totally different trend to the ADC and ODC activity. The GAD activity with SNAP treatment showed non-significant changes and was at its lowest level at 48 h, compared to the three other treatments. The GAD activities of PTIO, L-NNA and control treatments decreased by 12 h, and the L-NNA treatment reach its lowest value at 24 h. It is worth mention that the GAD activity of the L-NNA treatment was always the lowest of the treatments, being 72.8%, 32.3% and 11.1% lower at 12 h and 77.6%, 55.7% and 40.0% lower at 24 h compared to the SNAP, control and L-NNA treatments, respectively. Then these other three treatments gradually increased and reach their maxima at 48 h.

The GABA contents (Fig. 1C) of SNAP, L-NNA and PTIO treatments were 14.3%, 32.3% and 37.6% lower than the control at 12 h and 45.6%, 36.8% and 28.2% lower at 24 h. The GABA content of the SNAP and L-NNA treatments decreased in the first 24 h. In PTIO treatment, the GABA content decreased in the first 12 h, and then gently increased. In the control treatment, the GABA content decreased in the first 12 h, then remained at the same level until 48 h. The GABA-T activity (Fig. 1D) showed an interesting trend. The GABA-T activity of the SNAP treatment rose gradually until 48 h and decreased moderately with time under PTIO treatment. The GABA-T activity of L-NNA treatment showed a different trend with an increase in the first 12 h and then remained constantly until 24 h, and finally increased at 48 h. The control exhibited a similar trend to the SNAP treatment, but the activity was 9.6% lower than for the SNAP treatment at 24 h.

### PAs contents

**Figure 2:**
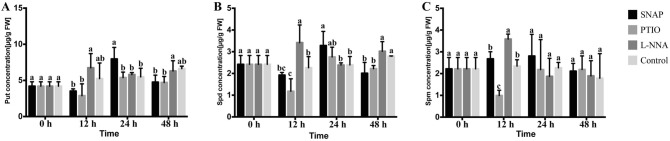
Changes in concentration of polyamines (PAs) during the different treatments. Value are means ± SE (n = 3). Means with different letters significantly differ from each other (p ≤ 0.05).

The UPLC measurements of Put, Spd and Spm showed relative stable contents in roots with cold treatment. The contents of Put (Fig. 2A) and Spd (Fig. 2B) with SNAP treatment were significantly higher compared to the control at 24 h. The Spd and Spm concentrations of PTIO treatment decreased rapidly at 12 h. Interestingly, after applying L-NNA, the Spd and Spm (Fig. 2C) concentrations were obviously higher than for the other three treatments at 12 h. The Put and Spd concentrations were significant higher with L-NNA treatment than the SNAP and PTIO treatments at 48 h. In addition, there were no significant differences among the four treatment groups at 48 h.

### NOS activity

**Figure 3:**
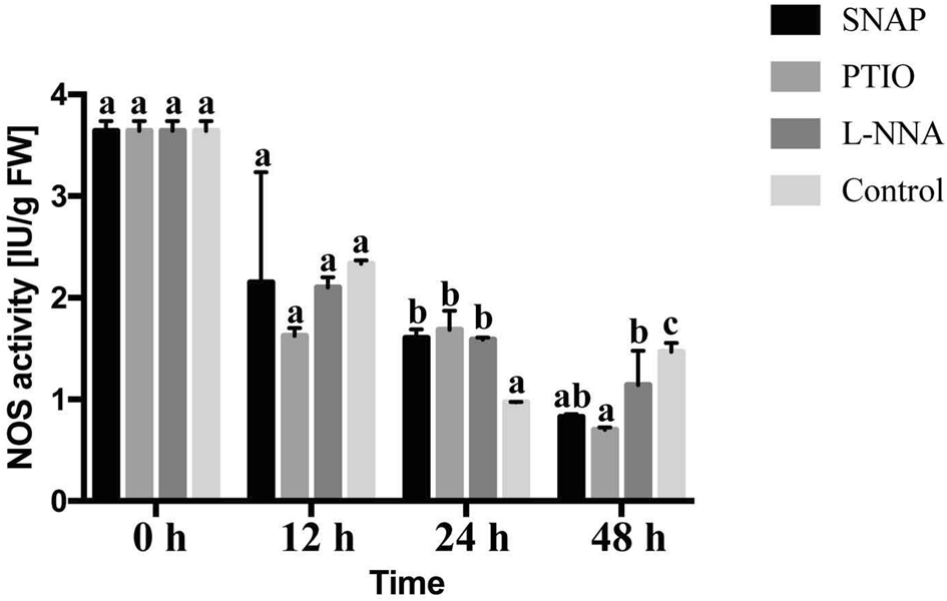
Changes of NOS activity during the different treatments. Values are means ± SE (n = 3), p ≤ 0.05.

The NOS activity of the four treatments showed a downward trend, but NOS activity of the control rose after 24 h (Fig. 3). In the first 12 h, the NOS activity of the PTIO treatment greatly decreased, and then maintained this level at 24 h, and gradually decreased by 48 h. At 12 h, the PTIO treatment NOS activity was 30.4% lower than the control value. The NOS activity of the control reach the bottom and was 73.5% lower than PTIO treatment. It’s totally different from the value at 12 h. The NOS activity of SNAP and L-NNA treatments showed no significant changes at the three time points, with the two trends essentially overlapping.

### Proline metabolism

**Figure 4:**
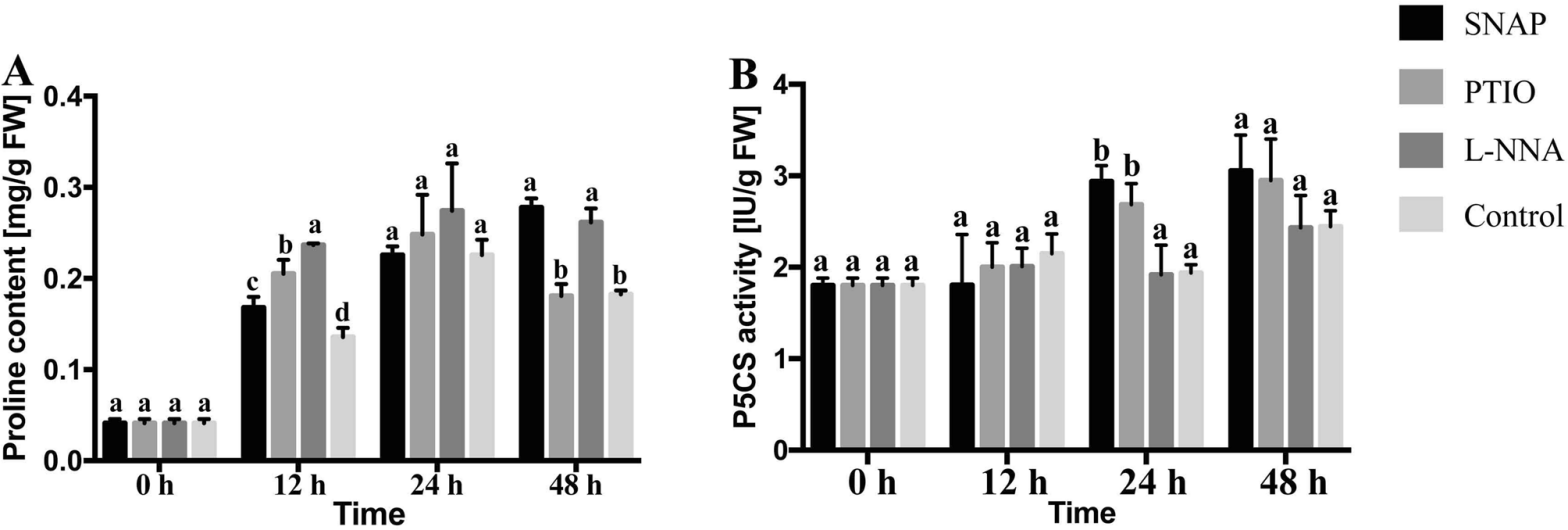
Activity of P5CS and proline content during treatments. Values are means ± SE (n = 3). Means with different letters significantly differ from each other (p ≤ 0.05).

Cold stress resulted in higher proline contents in tea roots for the four treatments (Fig. 4A), and progressively increased in the first 24 h, the value in the L-NNA treatment was always the greatest. At 12 h, proline contents of L-NNA, PTIO and SNAP treatments were 74.2%, 51.0% and 23.7% higher than the control, respectively. An interesting phenomenon occurred at 48 h, the proline content of the PTIO treatment suddenly decreasing while that for the SNAP continued to increase, as for L-NNA treatment, the proline content was remained at the same level. The control showed a moderate decrease and at the same degree as the PTIO treatment.

The P5CS activity (Fig. 4B) for the SNAP treatment showed non-significant changes in the first 12 h but increased for the control treatment. Then there was a unique response of P5CS activity of the SNAP treatment suddenly increasing, while the control showed a gentle decrease. The P5CS activity of L-NNA treatment had a continuous rising trend. The P5CS activities for SNAP and L-NNA were 16.0% and 7.0% lower than the control, respectively; however, at 24 h, the values were 51.5% and 38.6% higher than the control. For the PTIO treatment, the P5CS activity was about 6.6% lower than the control at 12 h, but there were non-significant changes at other time points.

### qRT-PCR analysis

**Figure 5:**
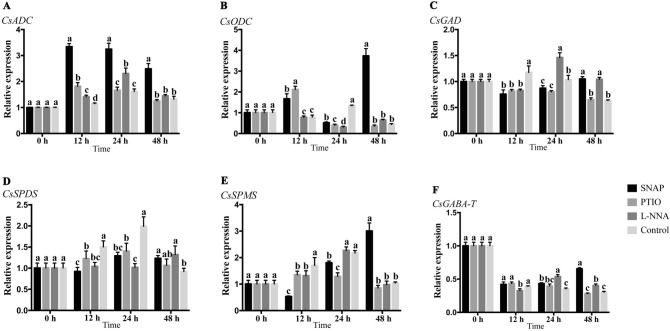
Expression analysis of related genes (*CsADC*, *CsODC*, *CsGAD*, *CsSPDS*, *CsSPMS*, and *CsGABA-T*) during the treatments using qRT-PCR. Values are means ± SE (n = 3). Means with different letters significantly differ from each other (p ≤ 0.05).

Expressions of related genes were determined using qRT-PCR to investigate the effects and relationships for each treatment. The *CsADC*, *CsODC*, *CsSPDS* and *CsSPMS* are the key genes of PAs biosynthesis. The *CsADC* was essentially up-regulated by SNAP and reached a maximum about 2.9-fold compared to the control at 12 h. The *CsADC* expression (Fig. 5A) was gently up-regulated by PTIO treatment at 12 h and then remained at the control level until 48 h. The L-NNA treatment resulted in up-regulation of *CsADC* at 12 h and 24 h, which then declined to the same level as the control.

The expression of *CsODC* exhibited a strange trend (Fig. 5B). the SNAP treatment enhanced its expression in the first 12 h then weakened it at 24 h; however, the expression reached a maximum about 8.6-fold than control at 48 h. The PTIO treatment resulted in a similar trend for *CsODC* expression, but the higher expression at 12h. The L-NNA treatment maintained the same level as the control at 12 h and 48 h, but had low expression at 24 h. Uniquely, at 12 h and 48 h, the *CsODC* expression in the control was lower or equal to the other treatments, but the expression at 24 h was much higher than three other treatments.

The *CsSPDS* and *CsSPMS* are related to PAs metabolism by catalyzing the conversion of Put to Spd and Spd to Spm, respectively. The *CsSPDS* expression (Fig. 5D) of the control was higher than the other treatments before 24 h, and reached a maximum at 24 h, almost 1.53-, 1.42- and 1.94-fold compared to the SNAP, PTIO and L-NNA treatments, respectively, then declined sharply below the other treatments. The SNAP treatment showed a slight increase in *CsSPDS* expression at 24 h and 48 h; additionally, expression of *CsSPDS* for the PTIO treatment also showed small increases at 12 and 24 h, and there was a slight rise for L-NNA treatment at 48 h.

The control and the PTIO treatment showed the same trends for *CsSPMS* expression (Fig. 5E). Interestingly, SNAP treatment strongly affected the expression of *CsSPMS*, which declined at 12 h and then rose until 48 h, the maximum value was 2.9-fold compared to control.

Glutamate is the main precursor of GABA, and *CsGAD* is the regulator of enzyme GAD. The *CsGAD* expression (Fig. 5C) in the control was higher than three other treatments at 12 h and then declined until 48 h. At 24 h, the L-NNA treatment caused high expression of *CsGAD*, but the expression of SNAP and PTIO treatments remained at the same level and was lower than L-NNA treatment. However, at 48 h, the *CsGAD* of L-NNA treatment down-regulated compared to 24 h, and the *CsGAD* of SNAP treatment at 48 h was up-regulated compare to 24 h.

Enzyme GABA-T catalyzes a reversible conversion between succinic semialdehyde and GABA. Expression of *CsGABA-T* (Fig. 5F) rapidly declined by half in the first 12 h at the same level. Then the SNAP treatment moderately rose with time but remained below the initial level. The PTIO treatment caused the *CsGABA-T* expression to decrease with time; however, for L-NNA treatment, expression of *CsGABA-T* gently rose to 24 h and then declined. In contrast, the qRT-PCR results according to the GABA metabolism correspond to the GABA content.

**Figure 6:**
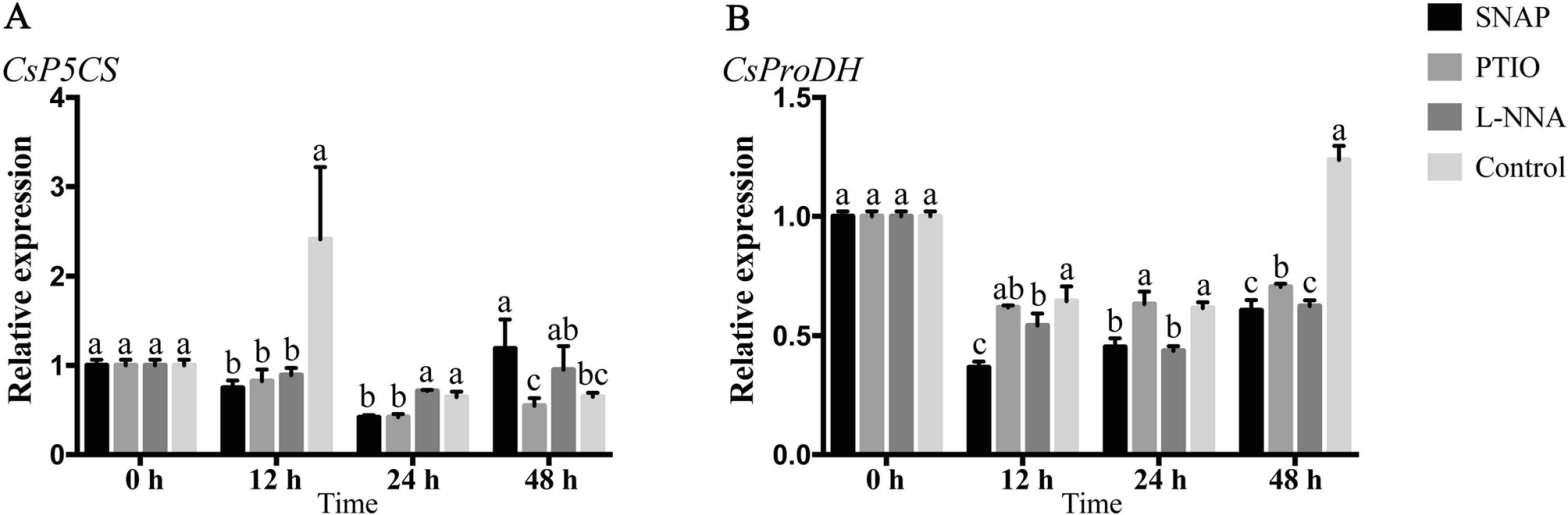
Expression analysis of *CsP5CS* and *CsProDH* during the treatments by using qRT-PCR. Values are means ± SE (n = 3). Means with different letters significantly differ from each other (p ≤ 0.05).

The enzyme P5CS is the key enzyme which related to proline biosynthesis. We determined *CsP5CS* (Fig. 6A) expression for all four three treatments. We gained the unexpected results that all the data exhibited a lower expression level except the control show extremely rise at 12 h. The SNAP treatment resulted in a gentle decrease until 24 h, which then rose, but remained below the initial level. Pyruvate dehydrogenase (ProDH) is a well-known enzyme associated with proline catabolism. The SNAP, PTIO and L-NNA treatments resulted in down-regulation of *CsProDH* (Fig. 6B). However, expression of the control declined at 12 and 24 h and then rose rapidly to reach the initial level at 48 h. The expression of *CsProDH* indicated that proline degradation was inhibited.

## Discussion

**Figure 7:**
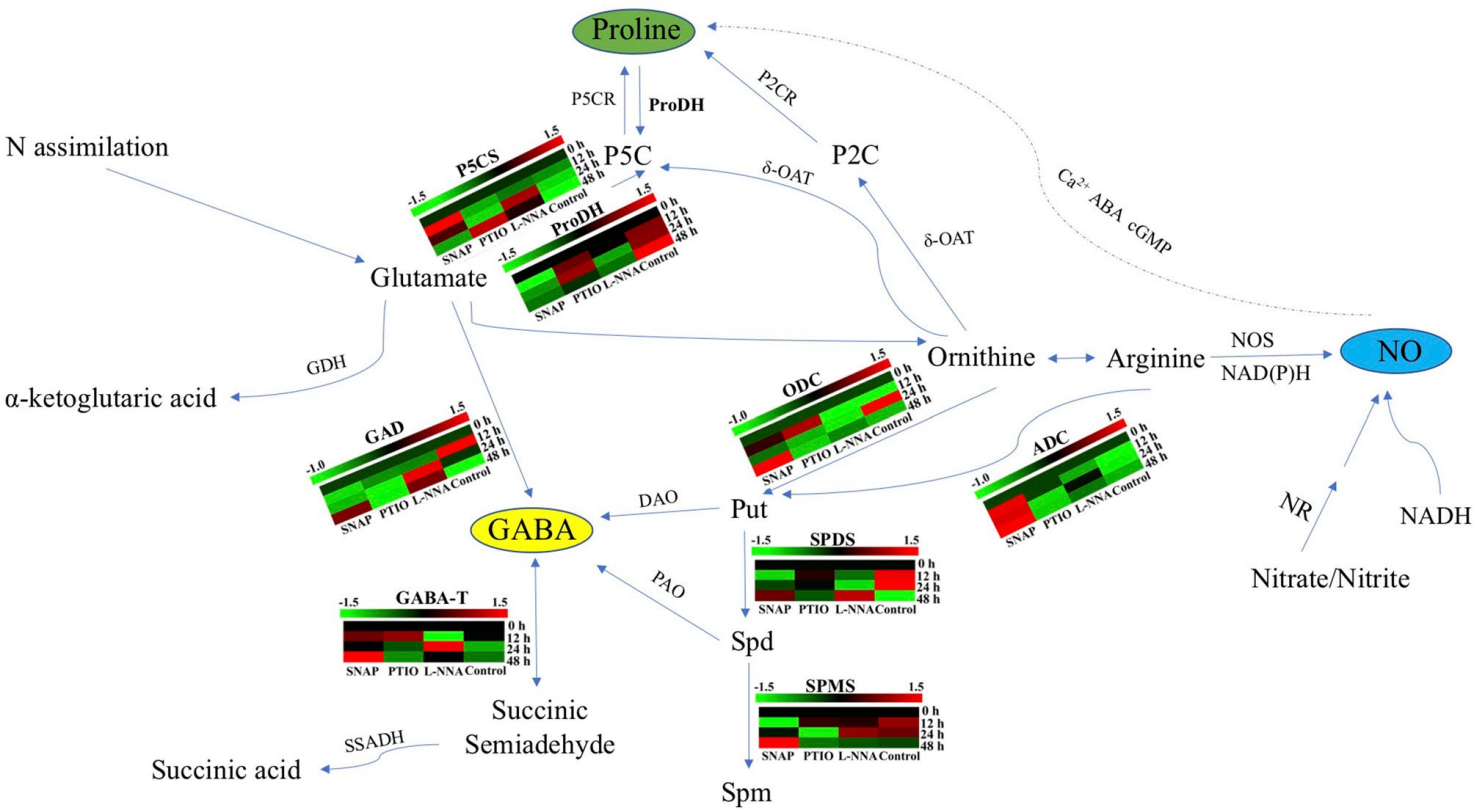
The proposed connections among GABA shunt, PAs synthesis and NO constitute major interactions in tea plants. Expressions of each gene were presented by heatmap, the value of the heatmap is log_2_ expression-value, the values were under row scale. The heatmap images were generated by TBtools (V.0.6696 for Mac) (https://github.com/CJ-Chen/TBtools).

The connections among GABA shunt, PAs synthesis and NO constitute major interactions in plant defense (Fig. 7). The NO has been shown to be important in the plant transduction pathway, where NO can interact with other plant molecules, such as cyclic nucleotides (cAMP and cGMP), cytosolic calcium, hydrogen peroxide, brassinosteroids and abscisic, jasmonic and salicylic acids^35–37^, which involve regulation of responses to biotic stresses such as salinity, drought, extreme temperature and heavy metal^38–40^. Their involvement in regulating cold stress in plants has been reported in diverse species, including *Arabidopsis thaliana*^24^ and *Populus trichocarpa*^41^. In this study, exogenous NO improved the proline content and related enzymes activities, shown it spotential ability to improve the cold resistance of tea plants.

It is well known that GABA is a signal molecule for regulating defense against various biotic and abiotic stresses^42,43^. Previous study revealed high accumulation of GABA in *Glycine max* (L.) Merr. roots under 6 days of salt stress^44^. Enzymes GAD and GABA-T work separately in catalyzing glutamate to GABA and its catabolism in plants. According to Wang et al.^45^, GABA content continues to rose over a period of 56 days in bamboo shoot. Our study showed the different results, which causing the drop of GABA content under cold stress within 48 h according to the control. The rising GAD activity with SNAP treatment did not result in higher accumulation of GABA. Moreover, *CsGABA-T* expression decreased after cold stress, but at 48 h, the expression of *CsGABA-T* of SNAP treatment was higher than control. We speculate that the GABA content has not risen to a significant level due to the limitation of processing time. However, there has been limited research exploring the response of plants to different abiotic stresses on plant roots.

It is clear that PAs are involved in responses to various environmental stresses^46^. The PAs are synthesized by ADC and ODC from arginine and ornithine^47^. Put and Spd are the major precursors of GABA^3^. In this study, we found that exogenous NO resulted in significant increase of ADC activity and up-regulation of *CsADC*. The SNAP treatment resulted in Put accumulation, promoted ADC and ODC activities. With the high GABA-T activity, accumulation of GABA was at its minimum. These results indicate that exogenous NO could improve cold tolerance by regulating PA metabolism.

Proline has been studied widely as a general indicator that responds to various environmental stresses^48^, playing a critical role as an osmoregulatory solute to maintain cell osmotic balance and alleviated cellular redox potential^45^. The synthesis of proline is mainly through the glutamate and ornithine pathways^29^ and P5CS and δ-OAT are the main participating enzymes^31^. In addition, the ProDH involved in this metabolism is a proline catabolic enzyme. In the present study, the SNAP treatment enhanced the proline content and P5CS activity and up-regulated *CsP5CS* expression. These results showed that the SNAP treatment can protect plants by enhancing related enzyme activities and expression of *CsProDH*, causing the accumulation of proline. We also observed that the NO scavenger (PTIO) resulted in a lower proline content and lower expression of *CsP5CS* compared to SNAP at 48 h. This result suggested that NO as a signal molecule may play a specific role in proline synthesis.

There are numerous possible sources of NO. The reductive pathways that lead to NO production depend on nitrite, which is primarily produced from nitrate by NR^49^. Moreover, NO can be generated non-enzymatically as a by-product of denitrification, fixation of nitrogen and respiration^50^. It is well known that the key enzyme for NO generation in animals is NOS, whereas, the activity of NOS-like enzymes has been detected in plants which catalyzed L-arginine synthesis to NO^51,52^ . A previous study showed that the decrease in arginase activity, which catalyzes conversion of L-arginine to ornithine resulted in increased NO production, while up-regulation of arginase reduced the release of NO. Activity of NOS was detected in the present study, although compared with NOS inhibitor treatment, there was no significant difference in NOS activity when treated with the NO donor. A similar phenomenon was also found in rice^53^; however, after treatment with the NO donor, the NOS activity in rice root decreased. Previous research showed that aluminum ions (Al^3+^) can significantly inhibit NOS activity^54^. In addition, tea plants are well known to be Al-accumulating plants that grow well in strongly acidic soils containing high levels of Al^3+^^55,56^. Based on our findings and previous research results, we hypothesize that the NOS-induced NO generation pathway is not the main pathway of NO production in roots of tea, or that there is a feedback relationship between NOS and NO content.

## Conclusion

This study compared the activities of enzymes and genes expression of GABA shunt and proline biosynthesis in different conditions of the presence of NO, reveal the NO can improve the cold resistance of tea plant by improving the ADC, ODC activities and proline accumulation. But NO contribute little to the GABA shunt as well as the PA biosynthesis within 48 h. And we speculate that NOS does not play a major role in NO synthesis in tea plants.

## Methods

### Tea cultivar and reagent

One-year-old tea plants (*Camellia sinensis* cv. Baiye No.1) were transplanted from botanic garden in Nanjing Agricultural University (Nanjing, China). Then washed the roots of tea plants with water and cultivated in Hoagland nutrient solution (pH 5.6), and the growth conditions were maintained at 23 ± 2 °C and 70 ± 10% relative humidity, 12 h light and 12 h dark. The plants were cultured for 30 days until the plants grew new roots. The S-nitroso-N-acetyl-DL-penicillamine (SNAP), 2-(4-carboxyphenyl)-4, 4, 5, 5-tetramethylimidazoline-1-oxyl-3-oxide (PTIO) and N’-nitro-L-arginine (L-NNA) were purchased from Sigma-Aldrich (St. Louis, MO, USA). Hoagland nutrient salts were purchased from Coolaber (Beijing, China).

### Treatments

Reagents used for treatments were dissolved in Hoagland nutrient solution. The concentrations of SNAP (NO donor), PTIO (NO scavenger) and L-NNA (NO synthase inhibitor) were 750 μM, 200 μM and 300 μM, respectively. Put 5 tea plants with new roots into the erlenmeyer flask containing the corresponding reagents for treatment. The control treatment was pure Hoagland nutrient solution (pH 5.6). In order to avoid the degradation of reagents caused by light, the surface of the Erlenmeyer flask is wrapped with foil sheet. All treatments were carried out at a low temperature of 4 ℃ in illuminated incubator (70 ± 10% relative humidity, 12 h light and 12 h dark). Samples of roots were harvested after 12 h, 24 h and 48 h, respectively, immediately placed in liquid nitrogen and stored at—80 °C for subsequently analyzed.

### Determination of ADC, ODC, GAD, GABA-T, NOS, P5CS activities and GABA content

The tea roots were ground in liquid nitrogen and then the powder was homogenized in 0.1 mM sodium phosphate buffer (pH 7.4), with ratio of powder to phosphate buffer of 1: 9 (weight: volume). Then homogenates were centrifuged at 3,500 g for 15 min, the supernatant used for enzymatic assays and to determine GABA content.

The supernatant was used for ADC, ODC, GAD, GABA-T, NOS, P5CS and GABA assay. The detection of ADC, ODC, GAD, GABA-T, NOS and P5CS activity and GABA content was used by the matched Assay Kit (Nanjing Jiancheng Biological Engineering Co., Ltd.). The procedures were followed manufacturer’s manual. The microplate reader (BioTek Co., Ltd. USA) was using for the measurement of absorbancy.

### Determination of PA content

The methods for extraction of free PAs and UPLC analysis were as described by Zhu et al.^11^ with slight changes. In brief, 1 mL 5% (v/v) cold perchloric acid (PCA) was added in 0.2 g power sample of roots and kept on ice for 30 min. After centrifugation of 12,000 g for 20 min at 4 ℃, the supernatants were combined and filtered using 0.22 μm filter syringe. 200 μL filtrate was transferred to a tube containing 200 μL 2 N NaOH and 10 μL benzoyl chloride. After 30 min incubation at 25 °C, 2 mL saturated NaCl was added. Then 2 mL of diethyl ester was added and thoroughly mixed, followed by centrifugation at 3,000×*g* for 5 min. Then, the upper phase was transferred to a new tube and dried under vacuum and the residue was resuspended in 100 μL methanol. The analysis method was same as Zhu et al.^11^.

### Gene expression analysis in tea plant

Real-time quantitative fluorescence PCR was performed by Nanjing Jisi Huiyuan Biotechnology Co. Ltd. Quantitative real-time PCR (qRT-PCR) was performed to evaluate the expression levels of *CsADC* (TEA009777.1), *CsODC* (TEA032079.1), *CsSPDS* (TEA018701.1), *CsSPMS* (TEA020668.1), *CsGAD* (TEA024088.1), *CsP5CS* (TEA031333.1) and *CsProDH* (TEA027545.1). The genes were referred to tea genome^33^. The primers of *CsADC*, *CsODC*, *CsSPDS*, *CsSPMS*, *CsGAD*, *CsP5CS* and *CsProDH* were designed by Primer 5 software (Primier, Canada). The primers of *CsGABA-T* refer to Mei et al.^34^. Each 20 μL of the PCR reaction solution contained 10 μL of 2 × SYBR Premix ExTaq (TaKaRa, Kyoto, Japan), 10 ng of diluted cDNA and 0.5 μΜ of gene-specific primers. The *Camellia sinensis* actin gene(actin-F, 5′-AGGGTTCTGTCCACTATGC-3′; actin-R, 5′-GGACGAAGAGCCTTTGCTACCG-3′) was used as an internal control for the assays. Amplification procedures of the thermocycling were as follows: 95 °C for 30 s, then 40 cycles of 95 °C for 10 s and 60 °C for 30 s. 72 °C single-point detection signal.

### Statistical analysis

All experimental data are mean values of a representative experiment (three repeats) and shown as mean ± standard error (SE). All statistical analyses were performed with SPSS 17.0 for Windows, and significance determined by Duncan’s test and ANOVA.

## Supplementary information

Supplementary Information.
