# Supplementary material for: Effects of nitric oxide on the GABA, polyamines, and proline in tea (Camellia sinensis) roots under cold stress
Source: Sci Rep. 2020 Jul 22;10:12240. doi: 10.1038/s41598-020-69253-y (PMC7376168; doi:10.1038/s41598-020-69253-y)
Supplement: Supplementary file 1 — Supplementary Information. [file 41598_2020_69253_MOESM1_ESM.docx]

**Effects of Nitric Oxide on the GABA, Polyamines, and Proline in Tea (*Camellia sinensis*) roots under Cold Stress**

Yuhua Wang^1, #^, Fei Xiong^1, #^, Shouhua Nong^1^, Jieren Liao^1^, Anqi Xing^1^, Qiang Shen^2^, Yuanchun Ma^1^ & Wanping Fang^1^, Xujun Zhu^1, *^

1. College of Horticulture, Nanjing Agricultural University, No.1 Weigang, Nanjing 210095, Jiangsu, P. R. China

2. Institute of Tea Sciences, Guizhou Academy of Agricultural Sciences, Guiyang 417100, China

| Primers | 5’ 3’ |
| --- | --- |
| *CsADC-F* | ACCAATTTTCCGGCGTACCT |
| *CsADC-R* | AACGGCTTGACGGAGATGTT |
| *CsODC-F* | AGCATTCCTCGGTGTCTTGG |
| *CsODC-R* | AAGTCGTGAGGTTTACGCCA |
| *CsSPDS-F* | GGACAACAGTTCCTACATACCC |
| *CsSPDS-R* | GGCAGAACCACACCTCTTTAT |
| *CsSPMS-F* | TCTTTGAGTCGGTAGCAAAGG |
| *CsSPMS-R* | CCACTTGGGTATGTAGGAACTG |
| *CsGAD-F* | AGTGACATCCAGAAAGTCTTGCT |
| *CsGAD-R* | CACCATTAGTCTTCTTCCTACTGAG |
| *CsP5CS-F* | GTCTCCACAAGACAGGAGTAAA |
| *CsP5CS-R* | GTCTCCACAAGACAGGAGTAAA |
| *CsProDH-F* | AATCCAACCGCCACAGTTCT |
| *CsProDH-R* | GTCCACCATCGGCTCTATCG |
| *CsGABAT-F* | TAGTATGTTGGCACCATTCAC |
| *CsGABAT-R* | ACCATAGACCAGCGAGAG |

**Table S1. Primers used in the study of gene expression analysis.**
